# A Chemiresistor Sensor Array Based on Graphene Nanostructures: From the Detection of Ammonia and Possible Interfering VOCs to Chemometric Analysis

**DOI:** 10.3390/s23020882

**Published:** 2023-01-12

**Authors:** Sonia Freddi, Michele Vergari, Stefania Pagliara, Luigi Sangaletti

**Affiliations:** Surface Science and Spectroscopy Lab @ I-Lamp, Department of Mathematics and Physics, Università Cattolica del Sacro Cuore, Via della Garzetta 48, 25123 Brescia, Italy

**Keywords:** sensor array, graphene, chemiresistor, electronic nose, ammonia, gas sensors, principal component analysis, linear discriminant analysis, VOCs, graphene nanocomposites

## Abstract

Sensor arrays are currently attracting the interest of researchers due to their potential of overcoming the limitations of single sensors regarding selectivity, required by specific applications. Among the materials used to develop sensor arrays, graphene has not been so far extensively exploited, despite its remarkable sensing capability. Here we present the development of a graphene-based sensor array prepared by dropcasting nanostructure and nanocomposite graphene solution on interdigitated substrates, with the aim to investigate the capability of the array to discriminate several gases related to specific applications, including environmental monitoring, food quality tracking, and breathomics. This goal is achieved in two steps: at first the sensing properties of the array have been assessed through ammonia exposures, drawing the calibration curves, estimating the limit of detection, which has been found in the ppb range for all sensors, and investigating stability and sensitivity; then, after performing exposures to acetone, ethanol, 2-propanol, sodium hypochlorite, and water vapour, chemometric tools have been exploited to investigate the discrimination capability of the array, including principal component analysis (PCA), linear discriminant analysis (LDA), and Mahalanobis distance. PCA shows that the array was able to discriminate all the tested gases with an explained variance around 95%, while with an LDA approach the array can be trained to accurately recognize unknown gas contribution, with an accuracy higher than 94%.

## 1. Introduction

In the last few decades, being set at front-end in IoT (Internet of Things), gas sensors have become quite important in everyday life and therefore, the interest of researchers in their development has noticeably increased. Market demands have pushed scientists to put their efforts in the development of extremely sensitive, low cost, reliable, stable, highly selective, and with rapid response and recovery, single sensors [1,2], exploiting different materials, from metal oxide nanostructures [3,4,5] and semiconductor layers [6,7] to polymers [8,9] and carbon-based nanomaterials [10,11,12], as well as different working configurations, i.e., capacitors [13,14], field effect transistors [15,16], or chemiresistors [8,14].

For several applications, including breathomics, food quality tracking, or environmental monitoring, a single sensor’s high selectivity is particularly important, since the analyte under investigation should be recognized and detected among many interfering gases [17,18]. Additionally, some applications specifically required the capability of a device to simultaneously monitor different compounds [17,19].

For these application fields, the development of a single chemiresistor sensor may not be the ultimate solution, on one hand due to the difficulties to maintain a high selectivity in presence of many interfering gases [17,18,20], on the other hand because of the inability of a single chemiresistor sensor to detect more than one specific analyte at the same time [20,21,22,23]. In turn, the development of a sensor array, or electronic nose, is preferable and it has very recently increased in popularity [17,18,20,21,22,23]. Indeed, the sensors inside an array do not necessarily need to be highly selective, since statistical treatment, as well as chemometric methods based on pattern recognition and/or multivariate data analysis, could be exploited as an alternative resource to properly analyze the data whenever experimental data alone do not offer the necessary selectivity. The aim of such chemometric analysis is often to achieve discrimination in a 2-dimentional or 3-dimensional space of the analytes under investigation, looking for clustering of data in this space.

As mentioned, among the materials exploited to develop gas sensors and sensor arrays, carbon-based materials are of particular interest due to their outstanding electrical properties [24,25]. Carbon nanotubes have been largely studied as single sensors [26,27,28] and, even to a lesser extent, also as electronic noses [17,29], while, only in the past few years, graphene, thanks to its high sensitivity to the surface adsorption of gas molecules [30], its high in-plane conductivity, and its low electrical intrinsic noise [31], has attracted the interest or researchers working in the sensors field. Although recent studies have clearly demonstrated the possibility to develop highly sensitive graphene sensors after proper functionalization [32,33,34,35,36,37], still few are the works exploiting graphene to develop sensors array [38,39,40], especially in a chemiresistor configuration [41].

Furthermore, such studies often focus mainly on the sensing performances and disclosure of the sensing mechanism, neglecting a more in dept chemometric analysis on the data provided by the array.

The main scope of this work is to develop a chemiresistor graphene-based sensor array to investigate as a proof of concepts the possibility to exploit not only PCA for data discrimination, but to explore quantitative chemometric tools such as linear discriminant analysis (LDA) and LDA coupled with a Mahalanobis distance algorithm.

In this regard, an array composed of 5 graphene-based sensors on alumina/silver interdigitated substrates has been developed exploiting a very simple, easy, and low-cost technique, i.e., dropcasting from solution. In particular, solutions of graphene nanostructures and graphene nanocomposites have been considered: graphene dispersion (hereafter Gr dispersion), graphene nanoplatelets (hereafter Gr_nanoplatelets), Fe_3_O_4_/graphene nanocomposite (Gr_Fe_3_O_4_), CoPt/graphene nanocomposite (Gr_CoPt), and TiO_2_/graphene nanocomposite (Gr_TiO_2_). These materials have been selected with the aim of exploring and comparing their sensing properties when used in a chemiresistor configuration, and to check their capability to work in an array. All sensing layers are related to a common platform material, i.e., graphene, which allowed to diversify the single layers by morphological properties, in the case of graphene dispersion and graphene nanoplatelets, or through the nanoparticles used for graphene decoration (namely, two oxides, TiO_2_ and Fe_3_O_4_, and a metal alloy, CoPt).

After characterization through Raman spectroscopy, the prepared samples have been put on a properly designed platform, which is able to simultaneously monitor the response from all sensors and allows for a direct comparison of the sensor performances under the same working conditions. Since ammonia is often considered as a benchmarking molecule for novel nanocarbon-based sensors [42,43], exposures to this analyte have been at first carried out, drawing the calibration curves of the 5 sensors and disclosing a good sensitivity compared to literature results. Additionally, the stability of the responses to ammonia up to 3 months after the sample’s preparation has been disclosed.

The discrimination capability of the array against other gases has then been tested through exposures to acetone, ethanol, 2-propanol, water, and sodium hypochlorite, producing a dataset for chemometrics analysis. PCA performed on the dataset shows that the developed e-nose is able to discriminate all the tested gases. Furthermore, the contribution of each sensor in the discrimination is investigated and discussed, supporting the claims with experimental evidence. Finally, we demonstrated that with an LDA approach the array can be trained to accurately recognize each gas contribution, despite the limited dataset. Additionally, the Mahalanobis distance has been evaluated to offer an alternative approach for the identification of unknown data.

Incidentally, it is worth mentioning that all the tested gases are relevant for several applications, including breathomics, environmental monitoring, safety, and food quality tracking. Indeed, ammonia is recognized as one of the precursors of fine particulate matter, whose inhalation can provoke severe damage to human and animal respiratory systems [44]. Spoiled food, especially cheese and meat, releases small quantities of ammonia [45,46], while concentrations around 1 ppm of ammonia in the exhaled breath of a patient can be related to liver or kidney malfunctioning [47,48], and a very low concentration indicates chronic obstructive pulmonary diseases [49]. Recently, ethanol has started to be largely exploited in biofuel, due to its lower emission of carbon dioxide when compared to fossil fuels, but since it is not completely combusted in the car engines, it contributes to an increase in the smog [50]. Rotten fruits and vegetables release ethanol [51,52], whereas both ethanol and 2-propanol are identified as two of the lung cancer biomarkers [53]. Regarding acetone, it is released by rotten meat, spoiled bread, and, in general, carbohydrates [54], while in exhaled breath could be found in high quantities in diabetes and ketoacidosis patients [55,56]. Finally, children with cystic fibrosis exhaled high chlorine and sodium concentrations [57].

## 2. Materials and Methods

### 2.1. Sample Preparation

Graphene dispersion (1 mg/mL in DMF), graphene nanoplatelets (1 mg/mL, water dispersion), Fe_3_O_4_/graphene nanocomposite (10 mg/mL acetone dispersion, Fe_3_O_4_ NPs size: 5–25 nm), CoPt/graphene nanocomposite (10 mg/mL acetone dispersion, CoPt NPs size: 2–5 nm), and TiO_2_/graphene nanocomposite (10 mg/mL, acetone dispersion, TiO_2_ NPs size: 10–40 nm) have been purchased from Sigma Aldrich and used as received.

**Figure 1:**
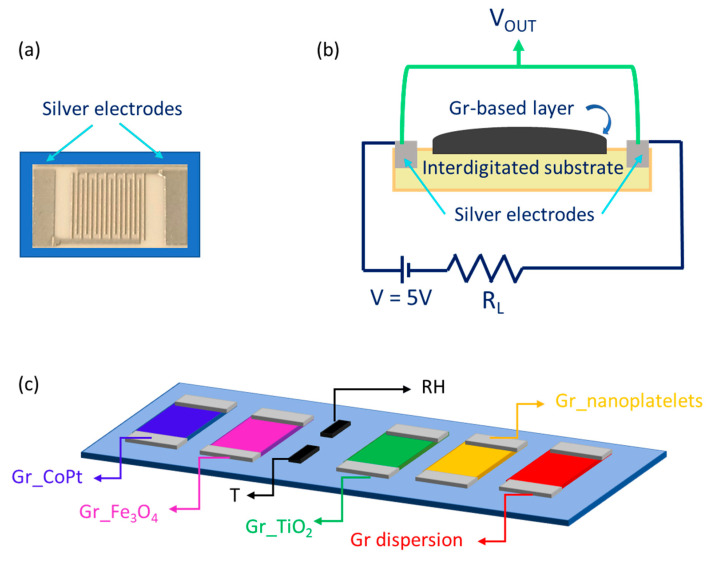
(**a**) A picture of the bare interdigitated alumina substrate with the silver electrodes; (**b**) the readout scheme of each chemiresistor sensor. (**c**) A schematic representation of the sensor array, assembled with the Gr dispersion (red), Gr_nanoplatelets (yellow), Gr_TiO_2_ (green), Gr_Fe_3_O_4_ (purple), and Gr_CoPt (blue). Relative humidity (RH) and temperature (T) commercial sensors are mounted in the middle of the array.

The size of silver interdigitates electrodes on alumina substrate was 7 mm × 13.4 mm. The interdigitates contacts consisted of 7 + 7 branches and each silver line has a 210 μm width. Figure 1a report the picture of an interdigitated substrate.

In order to prepare the sensors, 15 droplets of each graphene-based solution have been drop-casted on 5 interdigitated alumina substrates and let dry upon solvent evaporation at room temperature. The prepared samples will be labelled as Gr dispersion, Gr_nanoplatelets, Gr_Fe_3_O_4_, Gr_CoPt, and Gr_TiO_2_.

### 2.2. Sample Characterization

Raman spectra have been collected on the CGS device with a Renishaw-Invia system, equipped with a 532 nm laser source. The laser light has always been focused onto the sample with a 100× objective. An 1800 lines/mm grating and a laser power of 1 mW have been used for the measurements.

### 2.3. Gas Sensor Measurements

The five prepared samples have been mounted on a properly designed platform and they can work simultaneously (scheme in Figure 1c), allowing a direct comparison of the behaviours of the sensors under the same environmental conditions. Two commercial sensors have been put on the platform: a relative humidity (RH) sensor (humidity sensor HIH-4000 series—Honeywell Sensing) and a temperature sensor (Thermistor NTC PCB 5K—Murata).

The developed graphene-based array works in a chemiresistor configuration (Figure 1b): the gas analytes are detected by measuring the resistance changes of the sensing layers induced by the interaction with the gas molecules; the electronic circuit of each sensor comprises a load resistor (R_L_) in series with the sensor and by applying a constant voltage (V = 5 V) and monitoring the output voltage across the sample (V_OUT_), it is possible to track the resistance R of the sensor. The response of the sensor is then defined as ΔR/R_0_ = (R − R_0_)/R_0_, where R_0_ is the baseline sensor resistance before the gas exposure, and ΔR= R − R_0_ is the resistance variation due to the interaction with the gas molecules.

Gas exposures have been performed at room temperature (T = (25 ± 4) °C) and with a relative humidity (RH) value around 50 ± 5%, in order to investigate the sensing behaviour of the array in a working condition close to the application environment. Indeed, both the temperature and humidity tested range could be considered suitable for application in environmental monitoring, safety, and food quality tracking, where the array would work in STP conditions.

The recovery is always achieved in the same condition, fluxing humid air on the array. A scheme of the set up for the gas exposure is reported in Figure S1. The gas analyte, contained in certified cylinder (S.I.A.D. S.p.A.), is fluxed in a sealed homemade chamber (V = 1 L) through a mass flow controller (MFC) (MKS Instruments) connected to the sealed chamber. The MCF connected to the air cylinder has a maximum flow of 500 sccm, while the maximum flow of the MFC connected to the analyte cylinders is 200 sccm. In all the gas measurements, air has been used for both chamber purges after the exposure and to dilute the analyte in order to obtain different concentration exposures. Exposure time has been set at about 3 min. Considering several exposures, calibration curves for each sensor could be obtained by plotting the sensor response ΔR/R_0_ versus the gas concentration.

A commercial sensor (Figaro, Mod. TGS 2602) has been exploited to monitor and evaluate ammonia concentration. The reliability of this sensor calibration curve has been cross-checked by exposing the sensor in a testing chamber with calibrated mass flow controllers (MFCs) and certified ammonia and air cylinders.

### 2.4. Chemometric Analysis

To assess the discrimination capability of the array, the data collected from exposures to six different gases have been analyzed at first with principal component analysis (PCA) and then with linear discriminant analysis (LDA) and Mahalanobis distance, all implemented into the R software.

PCA is a chemometric method, which is unsupervised and can reduce the problem dimensionality while maximizing the variance of the data; it reorganizes the data into a set of principal orthogonal components (PCs), where PC1 is the component that has the greatest variance, PC2 represents the component with the second greatest variance, and so on. The aim of this statistical tool is to obtain a 2-dimensional or 3-dimensional space where a clear discrimination of the tested gases could be identified [58,59].

LDA is a supervised chemometric tool, therefore it takes into account the class properties of each sample; it assumes that each class has an identical covariance, and unlike the PCA, it deals only with the covariance of the data matrix. The optimal clustering among different classes (i.e., in this case different gases) is obtained by maximizing the distance between classes and minimizing the scattering of the data inside each class, resulting in a reduction of the problem dimensionality, and obtaining a new space generated by new coordinates, called discriminant factors [60]. LDA gives a better separation among different classes than PCA, allowing for an easier classification. Furthermore, it also allows for training and testing analysis on the dataset.

The Mahalanobis distance has been selected to evaluate the distance between a gas contribution and each cluster in an LDA space, with the aim to present an alternative approach for the identification of unknown data. Indeed, the Mahalanobis distance is the lowest for the class which a datum belongs to. The algorithm has been written in R as in ref. [61].

The data collected from the gas exposures have been pre-treated only by column mean-centering to feed the PCA, LDA, and Mahalanobis distance algorithm and a set of 32 exposures to six different gases has been considered.

## 3. Results and Discussion

**Figure 2:**
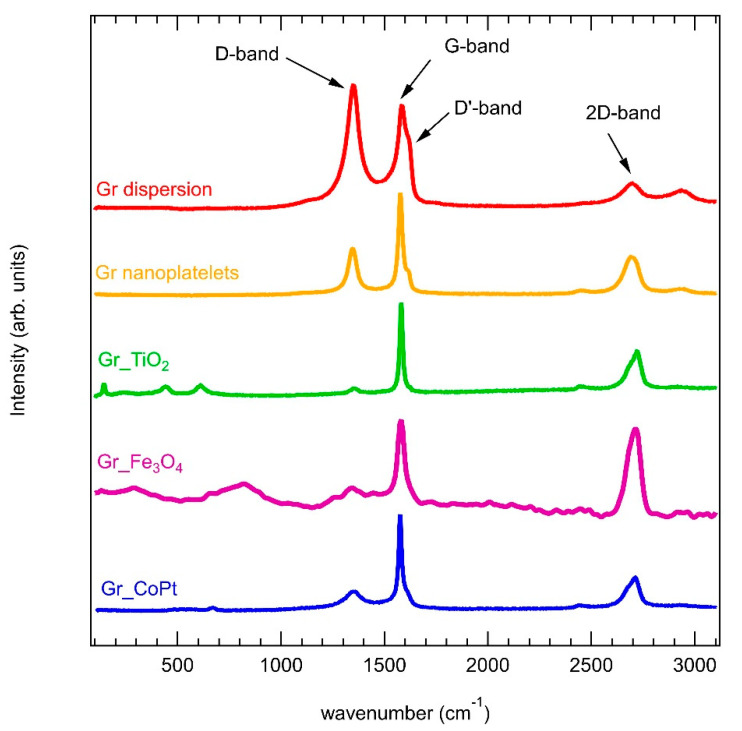
Representative Raman spectra of Gr dispersion (red), Gr_nanoplatelets (yellow), Gr_TiO_2_ (green), Gr_Fe_3_O_4_ (purple), and Gr_CoPt (blue) samples. All the spectra have been normalized at the G peak, in order to emphasize the presence of the D-band.

Figure 2 shows the representative Raman spectra collected on the five samples. In all spectra the typical peaks related to graphene are visible: the G-band is located at 1590 cm^−1^ and related to the C-C stretching of the sp^2^ atoms in the honeycomb lattice of graphene [62], and the 2D band is at around 2660 cm^−1^, a second order band related to the breathing mode of carbon atoms in the plane of graphene [62]. Regarding the 2D band, it is worth pointing out that the peak is not symmetric, as expected, due to the fact that the graphene flakes are randomly distributed on the alumina substrate after the dropcasting, and they do not form a uniform monolayer. The D-band, located around 1340 cm^−1^, can be ascribed to some disorder in the graphene structure [62], and it is present in all the samples, although its intensity is stronger in the Gr dispersion (red spectrum) and Gr_nanoplatelets (yellow spectrum) samples. A strong D-band presence corresponds also the appearance of the D’-band around 1615 cm^−1^, which is also related to the presence of defects [62].

Regarding the Gr_TiO_2_ spectrum (green curve), the contributions of the TiO_2_ NPs are visible at around 143 cm^−1^, 445 cm^−1^, and 615 cm^−1^, and in particular the peaks are related to the anatase and rutile phase, respectively [63,64]. The Fe_3_O_4_ contribution in the Gr_Fe_3_O_4_ sample (purple spectrum) could be found in the broad band at around 750 cm^−1^ [65]. Finally, for the Gr_CoPt spectrum (blue curve), a weak peak is present at around 680 cm^−1^, and it is related to the CoPt composites presence [66].

The I_D_/I_G_ intensity ratio in graphene resulted to be 2.70 ± 0.02, 0.73 ± 0.01, 0.13 ± 0.03, 0.44 ± 0.03, and 0.36 ± 0.02 for the Gr dispersion, Gr_nanoplatelets, Gr_TiO_2_, Gr_Fe_3_O_4_, and Gr_CoPt samples, respectively.

After the Raman characterization, the 5 samples have been set on a properly designed board, able to host all the 5 layers under investigation for the simultaneous detection of the sensor response during, at first, ammonia exposures.

**Figure 3:**
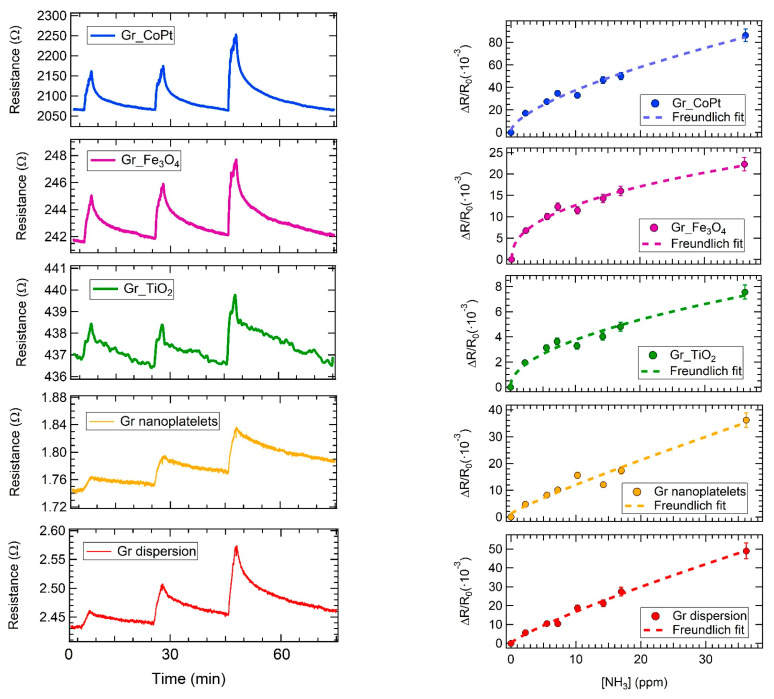
(**Left panel**): response of the sensor array to 14 ppm, 17 ppm, and 36 ppm ammonia exposures, respectively. (**Right panel**): calibration curves interpolated with a Freundlich fit; error bars represent standard deviation of the mean response obtained from three exposures to the same ammonia concentration.

As an example of the sensor array response, the resistance change measured upon exposure to three different ammonia concentrations (i.e., 14 ppm, 17 ppm, and 36 ppm) is shown in Figure 3 left panel. The exposure time has been set around 160 s.

First of all, all the sensors increase their resistance upon ammonia exposure, disclosing an overall p-type behaviour. Considering the recovery, defined as the time required for the sensor to return to 80% of the original baseline resistance after the gas exposure, it has always been achieved by all sensors at room temperature in about 15 min, Gr_CoPt, Gr_Fe_3_O_4_, and Gr_TiO_2_ being even faster.

In order to draw the calibration curves, several exposures to ammonia at different concentrations (from 2 ppm to 36 ppm) have been carried out and the results are reported in Figure 3 right panel. A Freundlich isotherm (∆R/R_0_ = y + A [NH_3_]^pow^) has been selected to interpolate the data and the fitting parameters, reported in Table S1, have been used to evaluate the limit of detection (LOD) according to the formula: 5σ/R_0_ = y + A [LOD]^pow^ [33,67], where σ is the fluctuation of the electrical signal. The evaluated limit of detection for all the samples is in the ppb range (Table S1) and in particular, the lowest values have been obtained for Gr_CoPt and Gr_Fe_3_O_4_: 0.1 ppb and 7.2 ppb, respectively. The higher values observed for Gr_TiO_2_, Gr dispersion, and Gr_nanoplatelets are mainly due to the larger fluctuations of the electrical signal (σ), which is noticeable in Figure 3 left panel.

The calibration curves present a sublinear behaviour, which is quite common in the field of gas sensor based on carbon nanomaterials [49,68,69].

Looking at the calibration curve, it is clear that the highest responses are obtained for the Gr_CoPt sensor (response range: 0–0.09), followed by Gr dispersion (response range: 0–0.05), Gr_nanoplatelets (response range: 0–0.04), Gr_Fe_3_O_4_ (response range: 0–0.025), and finally, the lowest response is detected by Gr_TiO_2_ (response range: 0–0.008).

The stability and reproducibility of the sensor response have been proven for ammonia detection and the results clearly demonstrated a good stability and reproducibility up to 3 months after the samples preparation (see supporting file, Figure S2).

Moreover, the stability of the sensors’ response to ammonia exposures in a working temperature range suitable for application in environmental monitoring, breathomics, and also food quality tracking (i.e., 21 °C < T < 29 °C) has been assessed (see supporting information file, Figure S3).

As for the sensing mechanisms, the resistance increase upon ammonia exposure indicates that the all layers exhibit an overall p-type nature, consistently with the fact that the electron injection from ammonia reduces the density of holes in these layers.

Regarding Gr dispersion and Gr_nanoplatelets layers, the sensing mechanism is well known in literature: an electron transfer from ammonia to graphene occurs when the N atom of ammonia faces the graphene lattice, as shown in Figure S4a, resulting in an overlap between the HOMO of ammonia and the graphene orbitals [70,71]. Furthermore, being that the graphene under investigation is not a perfect monolayer, it is possible to guess that the high number of defects, observed also by Raman spectroscopy, and edges present in the layer favors the interaction with ammonia [71,72].

Regarding Gr_Fe_3_O_4_ and Gr_TiO_2_ layers, although both Fe_3_O_4_ and TiO_2_ nanoparticles act as catalytic active centers, while graphene provides the conductive pathway, in addition to being itself a site for the interaction, the sensing mechanism is slightly different. In the case of Fe_3_O_4_, as reported in [73], the N lone pair in ammonia is supposed to donate electrons to the trivalent iron atom of Fe_3_O_4_ creating a pair of soliton electrons, while H atoms in ammonia interacts with the oxygen atoms in Fe_3_O_4_ forming a quite strong bond (see scheme in Figure S4b). Finally, as observed in other 2D materials functionalized with Fe_3_O_4_ NPs, these NPs allow for a fast electron transfer to the graphene layer [73].

Regarding the Gr_TiO_2_ sensor, two mechanisms have been suggested for ammonia sensing: (i) TiO_2_ is an n-type layer [74,75,76,77], when coupled with the p-type graphene, or in general with a p-type 2D-layer [77], it forms a p-n junction, which results in a depletion region positively charged on the TiO_2_ surface at the interface with the graphene layer, due to an electron transfer, and a consequent reduction of the activation energy for ammonia adsorption near the TiO_2_ surface (Figure S4c) [74,75]; (ii) TiO_2_ is considered as a Lewis’ acid and, being NH_3_ a Lewis’s base, a strong bond can be formed upon their interaction [76]. In both mechanisms, the electron exchanged between ammonia and TiO_2_ is easily transferred to graphene, and it is a reversible process [75].

Finally, no work has been published to date on gas sensors based on Gr_CoPt nanocomposites or CoPt NPs alone, therefore a sensing mechanism has not been proposed so far. It is possible to guess that the interaction with ammonia would occur in a twofold manner, as for Gr_TiO_2_ and Gr_Fe_3_O_4_ sensors: CoPt probably principally acts as a catalytic centre for ammonia interaction and then it will transfer the donated electron to the graphene layer (Figure S4d); at the same time ammonia can also interact with the graphene active sites.

Finally, a benchmarking with literature data has been performed considering the sensitivity parameter, defined as S = 100 × (ΔR/R_0_)/[NH_3_]. Of note, only papers based on graphene and clearly reporting gas concentration and sensor response/sensitivity, operating at room temperature and in a chemiresistor configuration have been taken into account for this benchmarking.

The comparison, reported in Figure S5, shows that the present results, obtained mainly in a low ammonia concentration range, are in line with the reported literature results on graphene chemiresistor sensors [32,33,78,79,80,81,82,83,84,85,86,87,88,89,90].

**Figure 4:**
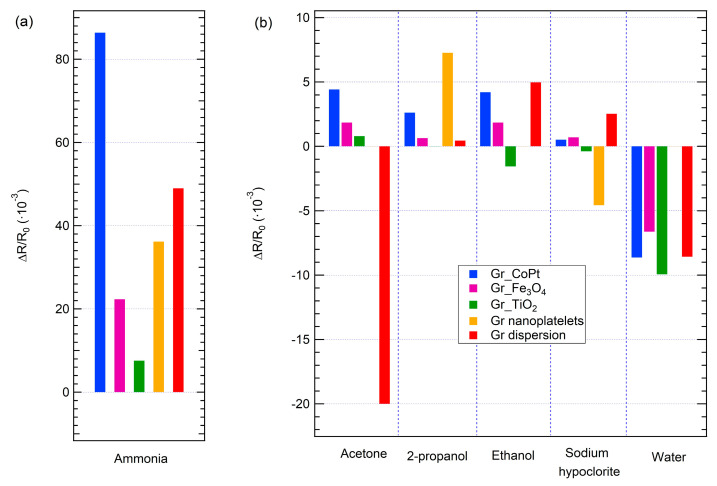
Response of the sensor array to selected target molecules: (**a**) ammonia; (**b**) acetone, 2-propanol, ethanol, sodium hypochlorite, and water.

As already mentioned, another important characteristic of single sensors is the selectivity, therefore, the prepared layers have been exposed to some of the most common interfering gases: acetone, ethanol, 2-propanol, sodium hypochlorite, and water vapours. Results are summarized in Figure 4, which reports the responses of the sensors array to the selected target molecules expressed as the sensor response ΔR/R_0_. First of all, the possibility to test the 5 sensors simultaneously allows for the identification of the best performing sensors: in this array, Gr dispersion shows a huge response to acetone as compared to the other sensors, while Gr_nanoplatelets appears to be a promising layer for 2-propanol detection.

Secondly, all the sensors change their resistance during exposure to all the tested gases, except Gr_nanoplatelets exposed to acetone, ethanol, and water and Gr_TiO_2_ exposed to 2-propanol; indeed, in these cases a resistance change has not been observed.

On the basis of these results, the present sensing layers do not display high selectivity: the extent of the response to ammonia (Figure 4a) is higher than the response to the other gases, as it is expected since the response to alcohols is usually low for carbon-based sensors [38,46], nevertheless the latter responses are not completely negligible (Figure 4b).

As often reported in literature [17,18,41] and already mentioned in the introduction, a way to overcome the selectivity problem for single chemiresistor sensors is to deal with an array coupled with chemometric analysis on the whole dataset collected with all chemiresistor sensors upon exposure to the selected target gas molecules at different concentrations, i.e., to deal with an electronic nose.

In order to enrich the data set, exposures to different concentrations of the same gas have been considered with the aim to perform PCA and LDA on the sensors’ responses.

In particular, a 5 × 32 response matrix has been used to perform chemometric analysis, with 5 sensors composing the array and 32 exposures performed at different concentrations of the six tested gases. The 32 exposures include 6 exposures to ammonia, 6 to water vapour, 5 to acetone, 5 to 2-propanol, 5 to sodium hypochlorite, and 5 to ethanol.

The concentration range is reported in Table S2.

**Figure 5:**
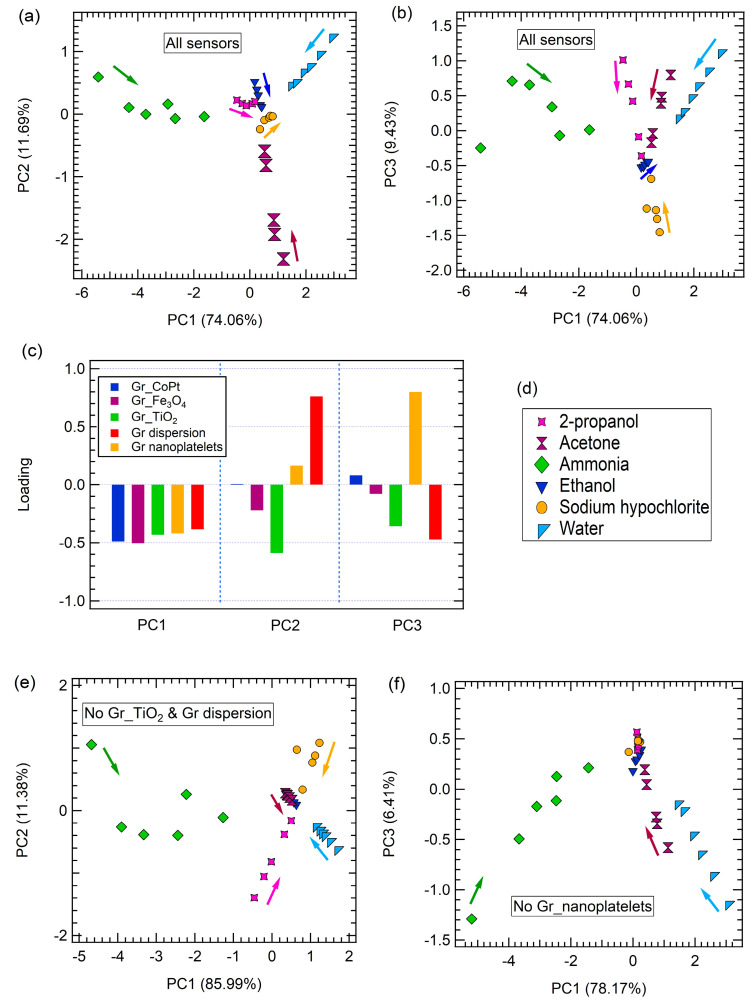
The PCA of the sensor array responses to exposure to ammonia, water, acetone, ethanol, and 2-propanol concentrations, performed considering all sensors: (**a**) PC2 vs. PC1, (**b**) PC3 vs. PC1, (**c**) loadings on PC1, PC2, and PC3, (**d**) legend of PCA contributions, (**e**) PCA result carried out without Gr dispersion and Gr_TiO_2_ sensors contributions, and (**f**) PCA space obtained without Gr_nanoplatelets sensor contribution. The arrows indicate the decreasing concentration trend.

The results of the PCA are shown in Figure 5. Considering the space generated by PC1 and PC2 (Figure 5a), as well as the space created by PC1 and PC3 (Figure 5b), all the tested gas molecules are clearly discriminated, since no overlap among different target gas clusters is observed. Furthermore, as previously found for arrays based on carbon nanomaterials [41,49], a concentration trend is established in each cluster, i.e., each gas concentration decreases going towards the center of the reference system, as indicated by the arrows in Figure 5. It is worth mentioning that although the developed sensors do not show a particular high response to all gases but ammonia, when their response is combined in the PCA analysis, the whole array is able to completely discriminate each gas contribution.

PCA loading plots can provide key information on the importance of each sensor in the discrimination capability of the whole array.

As shown in Figure 5c, which reports the loading of each sensor for each of the three components, all sensors equally contribute to PC1; therefore, they are all clearly involved in the discrimination of ammonia and water vapour from all the other gas contributions along the PC1 axis of both the PC1-PC2 and PC1-PC3 space. The second component is mainly defined by Gr dispersion and Gr_TiO_2_, respectively, while the Gr_nanoplatelets layer is the main responsible for PC3 discrimination. In particular, PC2 clearly separates acetone contribution from all the other gases, while along PC3 it is possible to discriminate sodium hypochlorite. It is worth noting that Gr_nanoplatelets and Gr dispersion sensors are the ones showing the best responses to sodium hypochlorite and acetone, respectively, and they are also the main responsible of their discrimination in a PCA space.

To further support the claim on the role of each sensor in the discrimination of a specific target gas, PCA has been performed removing from the dataset the response of the sensor that better define PC2 or PC3.

When removing Gr dispersion and Gr_TiO_2_ responses from the dataset, acetone is not discriminated anymore, but its contribution overlaps with ethanol (Figure 5e), whereas when removing Gr_nanoplatelets responses the discrimination of sodium hypochlorite on PC3 worsen considerably, resulting in an overlap between sodium hypochlorite and 2-propanol datasets (Figure 5f).

PCA alone is a chemometric tool that does not provide a probability on the output and, therefore, it is not possible to quantify its performance; indeed, PCA needs to be coupled to other algorithms, such as supported vector machine (SVM), in order to become predictive on the probability of a point to belong to a specific cluster [68].

On the contrary, LDA is a chemometric analysis that can be considered predictive and quantitative. In general, a predictive algorithm should be trained with an initial dataset (i.e., training dataset), which should provide the optimal linear combination of sensor results and the best separation between different clusters. In this context, LDA can be used to better separate the clusters related to the different gases, since it works maximizing the distance between classes and minimizing the scattering of the data inside each class.

After the training, a test dataset can be projected on the newly built model; in this way, unknown data can be recognized.

**Figure 6:**
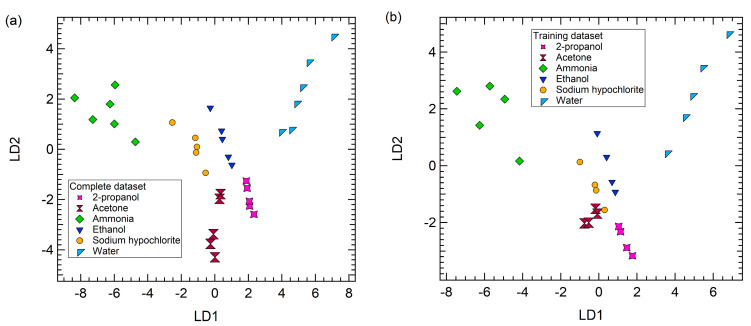
(**a**) The LDA space of the sensor array responses to ammonia, water, acetone, ethanol, and 2-propanol concentrations, performed considering the complete dataset (i.e., 32 exposures). (**b**) Example of the LDA space of the sensor array responses to ammonia, water, acetone, ethanol, and 2-propanol concentrations, performed considering a training dataset containing 26 exposures.

Performing LDA on the 32 exposures dataset, we also perform internal cross validation (CV) of the model with CV segment equal to 6; this mean that to build the model, the LDA algorithm randomly removes 6 datapoints and uses these data to test the newly built model, providing an accuracy of the model itself. Figure 6a shows the results of LDA performed on the whole dataset, which has been built with a cross validation accuracy of 100%.

After using the whole dataset to investigate the capability of the array to identify unknown data through LDA, we randomly split the dataset into two subgroups: a training and a test dataset, both containing data from the all the 6 tested gases, and we perform LDA on the training set only. The test set is then projected on the LDA direction to assess the capability of the array to correctly identify the nature of an unknown.

As an example, Figure 6b reports the results of the LDA performed on a training dataset that contains 26 data points, with a cross validation accuracy of 88% (see Tables S3 and S4 for the confusion matrix of cross validation and accuracy index); it is worth observing that the data point position in Figure 6b is not exactly the same of Figure 6a, since the LDA has not been carried out on the same dataset. 

After the LDA model has been drawn, the test dataset composed of 6 unidentified data (in this particular case one point from each gas class has been randomly selected to be part of the test dataset) has been used to validate the model. The model has been able to recognize and correctly assign each point to the correct class, as reported in Table S5, therefore the prediction accuracy is 100%.

**Figure 7:**
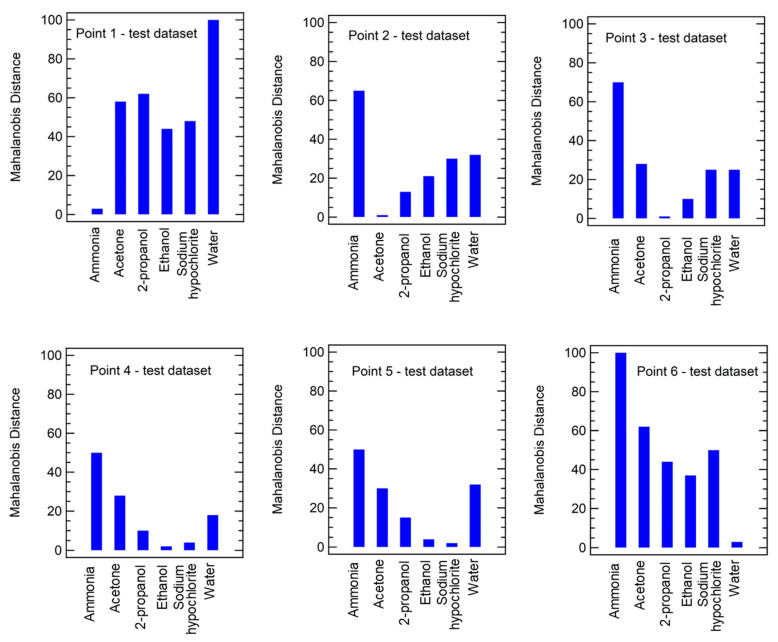
Example of Mahalanobis distances evaluated for each point of the test dataset containing 6 data as function of the 6 gas classes.

Moreover, after external validation it is possible to investigate the distance of each point in the test dataset from the different gas clusters, generated by the LDA model, and recognize the belonging class as function of the distance: the smallest distance identifies the class which the point belongs to. To carry out this task, Mahalanobis distance algorithm, being the most exploited for classification, has been considered and the results are reported in Figure 7.

In this example, it is clear that point 1 belongs to the ammonia cluster, point 2 to acetone class, point 3 to the 2-propanol class, and point 6 to water class. Regarding points 4 and 5, the smallest distances are for ethanol and sodium hypochlorite, respectively, but those points are quite close also to sodium hypochlorite and ethanol groups, respectively. This result is in agreement also with the LDA model presented in Figure 6b, where it is possible to notice that ethanol and sodium hypochlorite contributions cluster very closely.

Of note is also that the highest distance values are obtained considering point 1 and water and point 6 and ammonia; also in this case, this result is quite expected since point 1 belongs to ammonia cluster and point 6 to water group and ammonia and water contributions are quite distant in the LDA model (Figure 6b).

**Figure 8:**
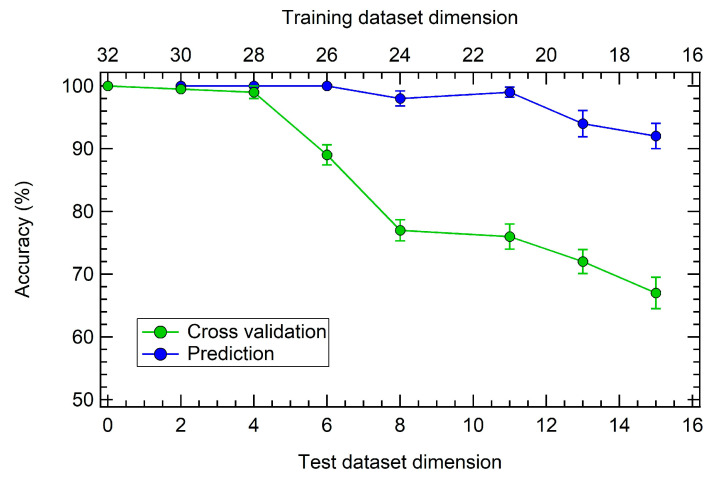
Accuracy percentage as function of the training-test datasets dimension for internal cross validation (green dots) and prediction (blue dots). Bottom axis: test dataset dimension (N, with 0 < N < 16). Top axis: training dataset dimension (32-N).

Finally, Figure 8 shows the accuracy of internal validation and prediction for several different combinations of training-test datasets dimension (i.e., 32-0, 30-2, 28-4, 26-6, 24-8, 21-11, 19-13, and 17-15), by picking 10 random subsets for each case. The internal CV ranges from 100% to 67%, while prediction accuracy goes from 100% to 94%.

The present results then support the application potential for this sensor array, even with a relatively small dataset, such as the one reported in this study. While PCA performs remarkably well in the discrimination of each gas contribution, for a practical application where prediction is required with specific accuracy, LDA should be preferred.

## 4. Conclusions

The goal of the present work was to develop a graphene-based sensor array in a chemiresistor configuration and to explore the discrimination capability of this array in the identification of different gases, related to specific applications, such as breathomics, environmental monitoring, and food quality tracking, exploiting few chemometric tools. The array has been prepared by dropcasting graphene nanostructures and graphene nanocomposites solutions on interdigitate alumina/silver substrates and after characterization, five different samples have been put on a properly designed platform. The goal has then been achieved in two steps: (i) ammonia exposures have been performed, the calibration curve of each sensor has been drawn, the limit of detection has been evaluated and it is in the low ppb range for all the sensors, a remarkable stability up to 3 months of all sensors response has been found and finally, the obtained results have been compared to literature, disclosing a good sensitivity for the present chemiresistors, which is in line with what has been so far published; (ii) exposures to acetone, 2-propanol, ethanol, sodium hypochlorite, and water have been carried out with the aim to prepare a dataset for chemometric analyses. These gases have been selected because of their interfering nature in many applications, including the ones for which the presented array is aimed to. In detail, at first it has been proven that the array can clearly discriminate the tested gases in a PCA space, considering both PC1-PC2 and PC1-PC-3 2D subspaces. Moreover, the role of each sensor in the discrimination is investigated and discussed, supporting the claims with experimental evidence. In particular, thanks to the loading plots, it has been found that all sensors are responsible for the discrimination of ammonia and water vapour, while Gr dispersion and Gr_TiO_2_ are required to discriminate acetone, and Gr_nanoplatelets is essential for sodium hypochlorite discrimination. The capability of the array to recognize unknown data with a high accuracy has been demonstrated by LDA, also investigating different training and test dataset dimensions; specifically, the prediction accuracy is always above 94%, when also halving the training dataset dimension.

Finally, an approach to identify unknown data has been suggested and tested by exploiting the Mahalanobis distance algorithm.

Further improvements in the ΔR/R_0_ response could be expected from the optimization of each layer thickness, as observed for instance in CNTs chemiresistors [67]. This is left for future studies as it goes beyond the scope of the present work.

The low-cost and simple preparation technique, as well as the easiness of use and simplicity of the sensor array itself, coupled with its remarkable discrimination and predictive capability, make the presented array suitable and promising for environmental monitoring, food quality tracking, and breathomics applications.

## Data Availability

The data presented in this study are available on request from the corresponding author.

## Supplementary Materials

The following supporting information can be downloaded at: https://www.mdpi.com/article/10.3390/s23020882/s1, Figure S1: Set up for gas exposures; Table S1: Freundlich fitting parameters and evaluated limit of detection; Figure S2: Stability of sensor response over time; Figure S3: stability of the sensor response at different working temperature; Figure S4: sensing mechanism; Figure S5: sensitivity benchmarking; Table S2: concentration range for all tested gases; Tables S3 and S4: confusion matrix for LDA cross validation with accuracy percentage evaluation; Table S5: confusion matrix for LDA predictive capability.
