# Supplementary material for: A Chemiresistor Sensor Array Based on Graphene Nanostructures: From the Detection of Ammonia and Possible Interfering VOCs to Chemometric Analysis
Source: Sensors (Basel). 2023 Jan 12;23(2):882. doi: 10.3390/s23020882 (PMC9862857; doi:10.3390/s23020882)
Supplement: Supplementary file 1 [file sensors-23-00882-s001.zip › sensors-2147139-supplementary.pdf]

Supplementary material to:

A Chemiresistor Sensor Array Based on Graphene Nanostructures: From the Detection of Ammonia and Possible Interfering VOCs to Chemometric Analysis

Sonia Freddi *, Michele Vergari, Stefania Pagliara and Luigi Sangaletti *

Surface Science and Spectroscopy Lab @ I-Lamp, Department of Mathematics and Physics, Università Cattolica del Sacro Cuore, Via della Garzetta 48, 25123 Brescia, Italy
* Correspondence: sonia.freddi@unicatt.it (S.F.); luigi.sangaletti@unicatt.it (L.S.)

Content:

1. Set up for gas exposures (Figure S1)
2. Freundlich fitting parameters and limit of detection (Table S1)
3. Stability of sensor response upon time and temperature. (Figure S2, Figure S3)
4. Sensing mechanism (Figure S4)
5. Sensitivity benchmarking (Figure S5)
6. Concentration range of each tested gas molecule (Table S2)
7. Example of a confusion matrix for LDA cross validation with accuracy percentage evaluation (Table S3, Table S4)
8. Example of a confusion matrix for LDA predictive capability (Table S5)

1. Set up for gas exposures

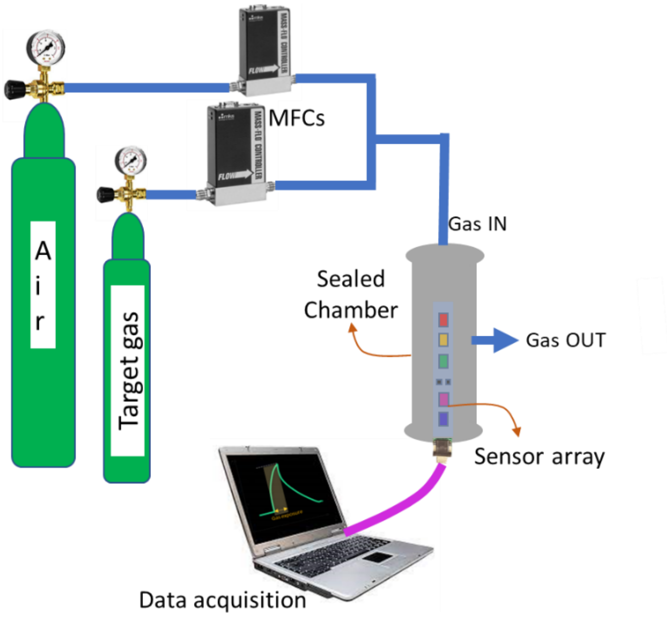

Figure S1: Schematic representation of the set up used for the gas exposures. The system comprises a sealed steal homemade chamber, connected to 2 mass flow controllers (MFCs) and a PC for data acquisition, a cylinder filled with air and a cylinder with the target gas molecules. The sensor array is hosted inside the chamber. The MCF connected to the air cylinder has a maximum flow of 500 sccm, while the max flow of the MFC connected to the analyte cylinders is 200 sccm.

2. Freundlich fitting parameters and detection limit evaluation

Table S1: fitting parameter of the calibration curve (reported in Figure 3 – right panel) of the main text), used to evaluate the limit of detection (LOD) for ammonia exposure, according to the formula reported in the main text.

**Sensor**	**A**	**Pow**	**LOD (ppb)**
Gr_CoPt	0.008 ± 0.001	0.65 ± 0.01	0.1
Gr_Fe_3O_4	0.005 ± 0.001	0.43 ± 0.01	7.2
Gr_TiO_2	0.0011 ± 0.0002	0.53 ± 0.03	101
Gr nanolapelets	0.0014 ± 0.0003	0.89 ± 0.04	720
Gr dispersion	0.0024 ± 0.0001	0.84 ± 0.01	102

3. Stability of sensor response upon time and temperature

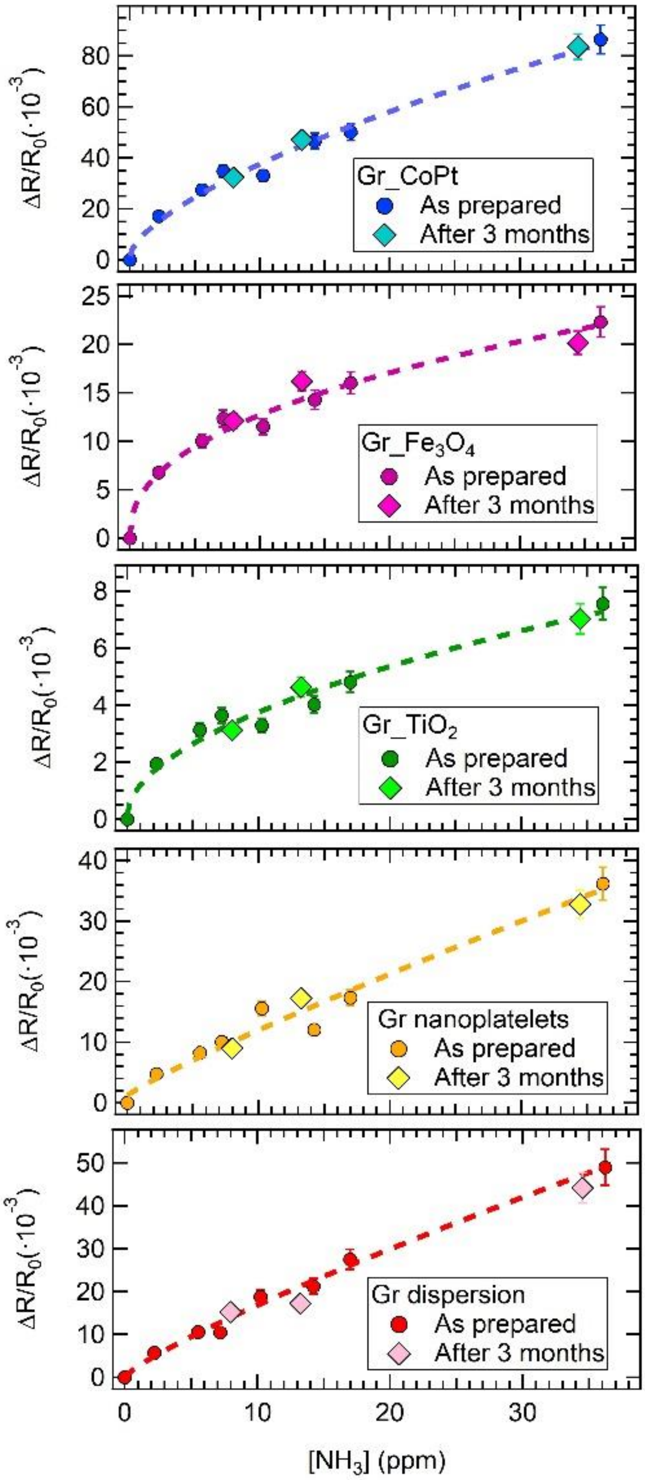

Figure S2: Proof of the stability and reproducibility of the sensors response upon ammonia exposures: calibration curves (dots) and Freundlich fit (dashed curves) evaluated after the sample preparation; rhombus data have been collected after 3 months from the sample preparation and they perfectly matched the calibration curve and Freundlich fit for all sensors.

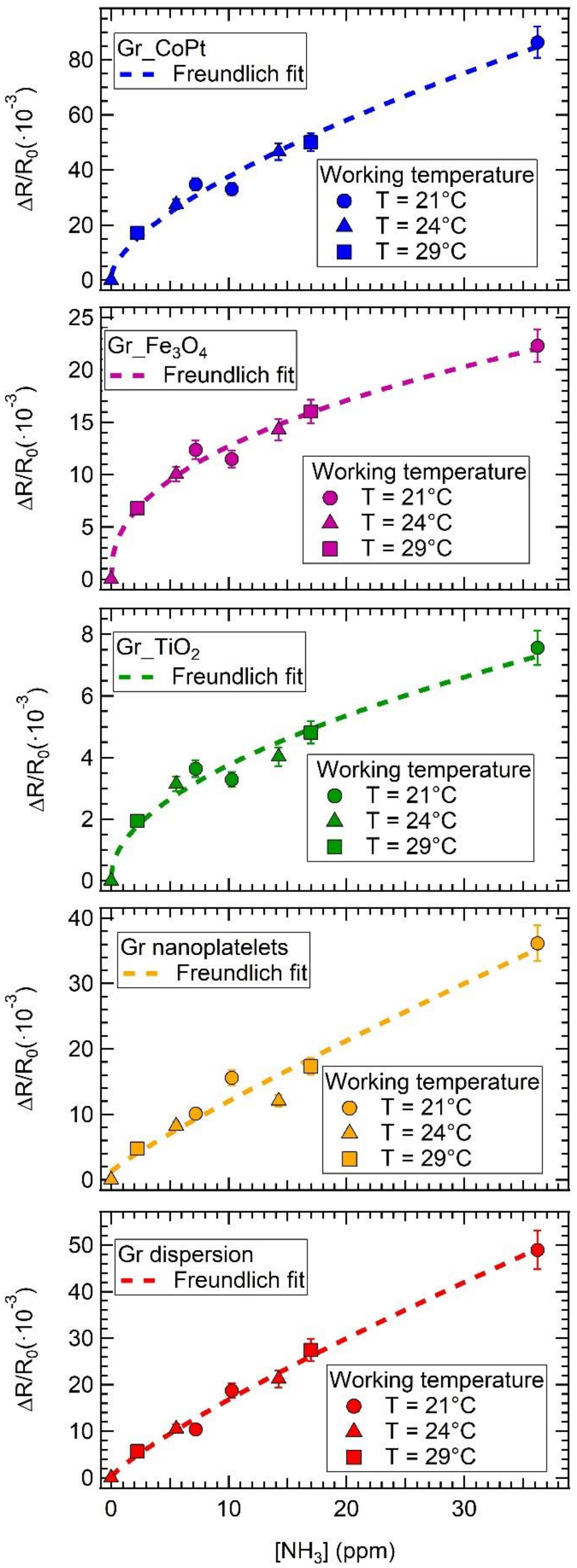

Figure S3: Proof of the stability and reproducibility of the sensors response to ammonia exposures at different working temperature: 21°C (dots), 24°C (triangles) and 29°C (squares). Dashed line is the Freundlich fit. The tested temperature range is suitable for breathomics, environmental, food quality and safety applications.

4. Sensing mechanism

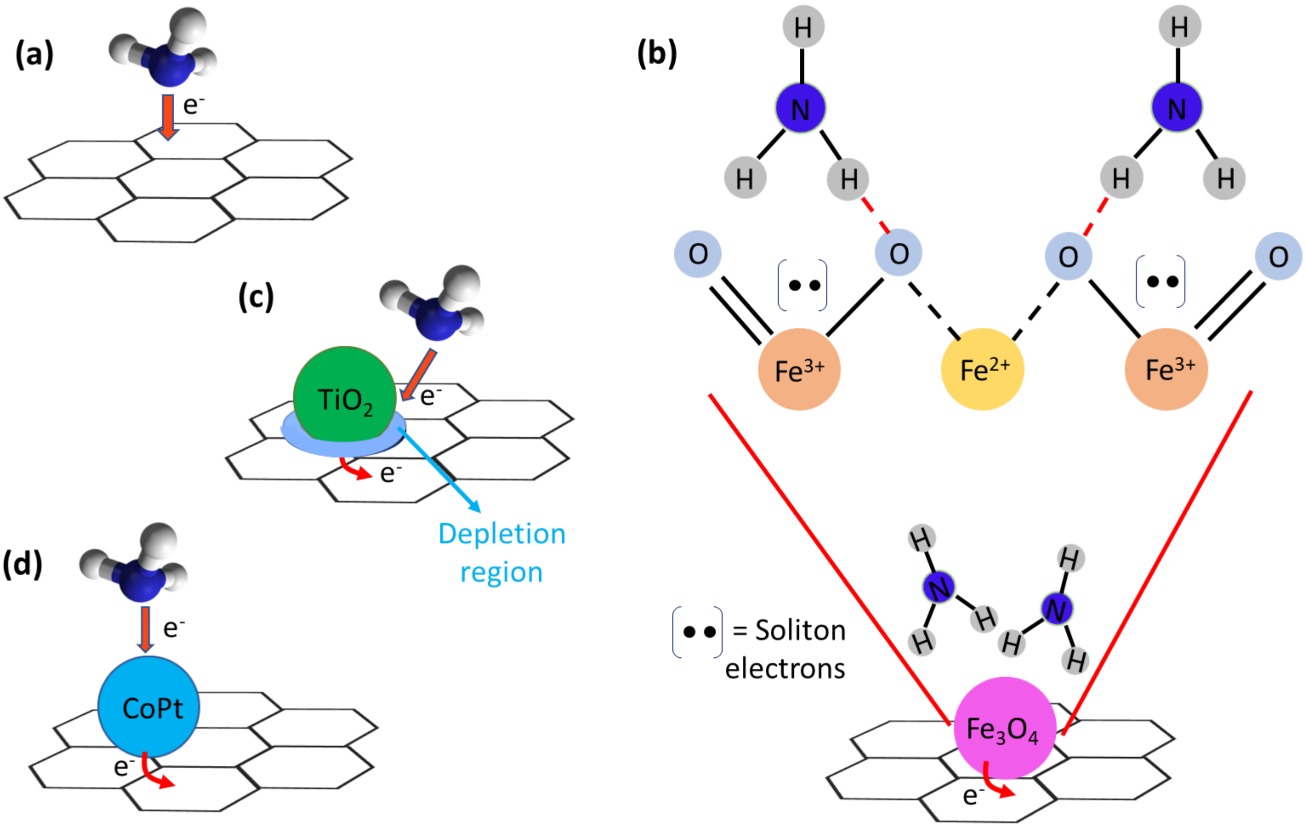

Figure S4: Scheme of the sensing mechanism to ammonia exposure for (a) Gr dispersion and Gr_nanoplatelets, (b) Gr_Fe_3O_4, (c) Gr_TiO_2 and (d) Gr_CoPt layers.

5. Sensitivity benchmarking

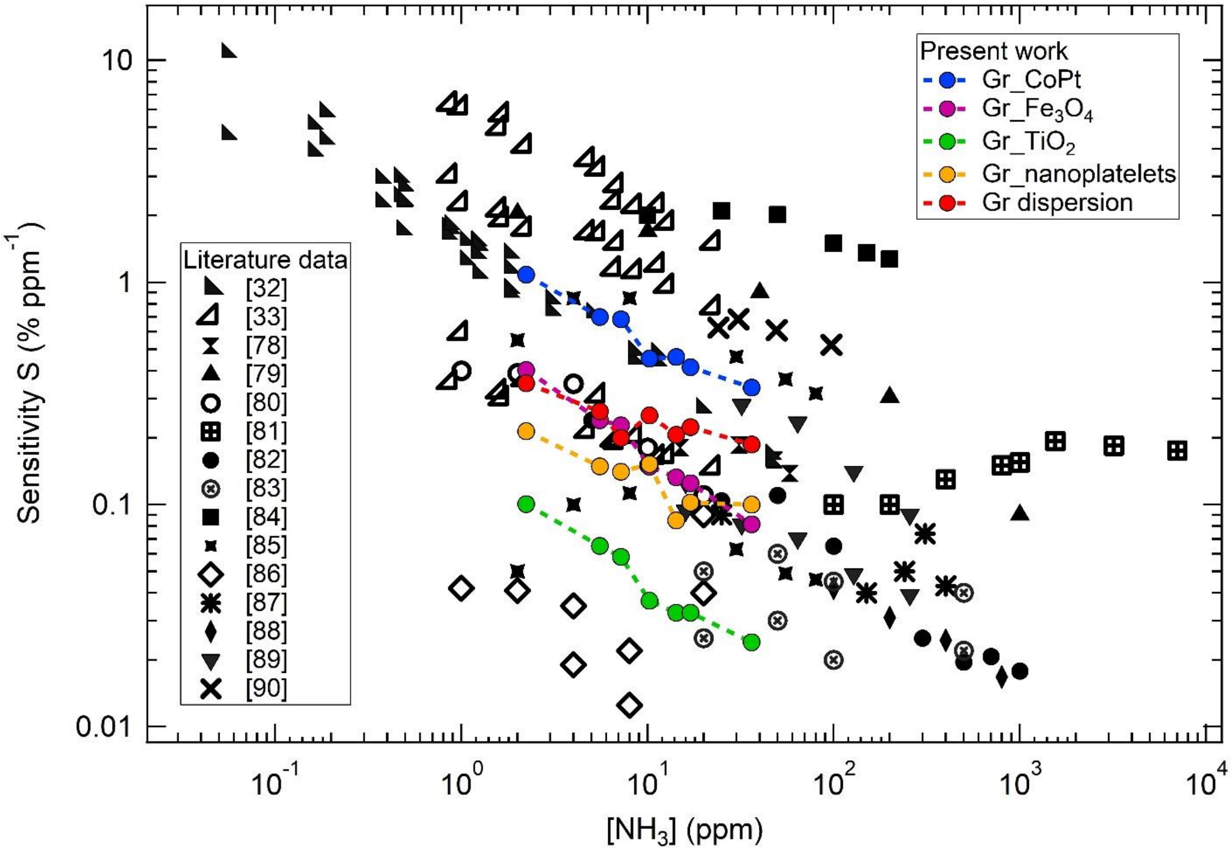

Figure S5: Benchmarking for sensitivity (defined as: $(\Delta R/R_0*100)/[NH_3]$) upon ammonia exposure, of the prepared sensors (blue, purple, green, yellow and red dots) with respect to other graphene-based chemiresistor sensitivity values reported in literature (black symbols). Literature data refer to Refs [32,33,78-90] reported in the main text. Data are presented in a log-log scale. Of note: only articles clearly reporting gas concentration and sensor response/sensitivity have been taken into account for this benchmarking.

6. Concentration range of each tested gas molecule

Table S2: Concentration range of the selected target gas molecules used for the PCA, LDA and Mahalanobis distance analysis.

Target gas	Concentration range (ppm)
Ammonia	0-36.0
Acetone	0-40.0
Ethanol	0-37.0
2-propanol	0-40.3
Sodium hypochlorite	0-0.5

7. <u>Example of a confusion matrix for LDA cross validation with accuracy percentage evaluation</u>

Table S3: Example of the confusion matrix upon internal cross validation of the model reported in Figure 6-b of the main text. On the raw there are the correct belonging classes, while the columns, labelled with an asterisk (*), are the predicted belonging classes. In red are enlightened the wrong assignment of data.

	**Ammonia***	**Acetone***	**2-propanol***	**Ethanol***	**Sodium hypochlorite***	**Water***
**Ammonia**	6	0	0	0	0	0
**Acetone**	0	3	0	0	1	0
**2-propanol**	0	0	3	1	0	0
**Ethanol**	0	0	0	4	0	0
**Sodium hypochlorite**	0	0	0	1	3	0
**Water**	0	0	0	0	0	5

Table S4: Accuracy percentage of cross validation referred to the confusion matrix reported in table S.III. The total accuracy is 88%.

**Ammonia**	**Acetone**	**2-propanol**	**Ethanol**	**Sodium hypochlorite**	**Water**
100%	75%	75%	100%	75%	100%

8. <u>Example of a confusion matrix for LDA predictive capability</u>

Table S5. Confusion matrix obtained for classification of a random subset of training dataset containing 6 data (one for each class) projected on the LDA model reported in Figure 6-b. The accuracy of this classification is 100% since all the data are correctly identified. True class is reported on row, while the predicted class are on the columns and labelled with an asterisk (*).

	**Ammonia***	**Acetone***	**2-propanol***	**Ethanol***	**Sodium hypochlorite***	**Water***
**Ammonia**	1	0	0	0	0	0
**Acetone**	0	1	0	0	0	0
**2-propanol**	0	0	1	0	0	0
**Ethanol**	0	0	0	1	0	0
**Sodium hypochlorite**	0	0	0	0	1	0
**Water**	0	0	0	0	0	1
